# Distinct effects of over-general autobiographical memory on suicidal ideation among depressed and healthy people

**DOI:** 10.1186/s12888-020-02877-6

**Published:** 2020-10-12

**Authors:** Wen Jiang, Guangtao Hu, Jingxuan Zhang, Ken Chen, Dongni Fan, Zhengzhi Feng

**Affiliations:** 1 School of Psychology, Army Medical University, Chongqing, China; 2 Department of Psychological and Psychological Medicine, The 958th Hospital of the Chinese People’s Liberation Army, Chongqing, China; 3 Outpatient of Chongqing Mental Health Center, Chongqing, China

**Keywords:** Autobiographical memory, Over-general memory, Depression, Childhood trauma, Suicidal ideation

## Abstract

**Background:**

Childhood trauma and over-general autobiographical memory (OGM) are crucial risk factors of suicide. This study aimed to investigate whether suicidal ideation was predicted by one’s childhood trauma and OGM and the mechanism of OGM underlying suicidal ideation in depression patients and healthy controls.

**Methods:**

A total of 180 depression patients and 176 matched healthy individuals were recruited in this study. Data were analyzed using descriptive statistics, and Pearson’s correlation coefficient was obtained. Path analysis was conducted to test a meditational model. The multigroup comparison was applied to find differences between groups.

**Results:**

Significant differences were detected between depression patients and healthy controls with respect to childhood trauma, OGM, suicidal ideation, and suicidal behavior. OGM was positively correlated with both current and worst-point suicidal ideation in the depression group and significantly correlated with worst-point suicidal ideation in the healthy control group. The path model showed that childhood trauma had a direct impact on the current suicidal ideation directly, and an indirect influence through OGM and worst-point suicidal ideation. Multigroup analysis further demonstrated that OGM affected and mediated the current suicidal ideation due to childhood trauma in depression patients, whereas only worst-point suicidal ideation was affected in healthy controls.

**Conclusions:**

The OGM mediates suicidal ideation in depression patients, but only affects the worst-point suicidal ideation in the healthy controls. As it is one of the major risk factors of suicidal ideation in depression, amelioration of OGM might be an useful method to reduce or prevent suicidal ideation in depression patients.

## Background

Suicide is a major public health concern worldwide. According to the report of the World Health Organization, one person commits suicide every 40 s. It has become the second leading cause of deaths among 15–29-year-olds globally [1]. The rate of suicide is much higher in depression patients than healthy people. About 65–80% of the depression patients have suicidal ideation, and 45–55% patients have suicidal or self-injury behavior [2]. Death by suicide is associated with a huge economic, social, and psychological burden for individuals, families, communities, and countries [3]. Thus, preventing suicides and clarifying suicide-related factors is an urgent requirement.

Suicidal behavior is a complex and progressive process. O’Connor et al. [4, 5] proposed and renovated the integrated motivational-volitional (IMV) model of suicidal behavior, dividing it into three phases: pre-motivational, motivational, and volitional. Firstly, the pre-motivational phase is composed of diathesis, environment, and life events, describing the background factors and triggering events. In this part, childhood trauma or early-life adversity is an unequivocal risk factor, which can disrupt attachment relationships, change the epigenetic inheritance of genes, and affect cortisol regulation [6]. Secondly, the motivational phase focuses on the psychological processes of suicidal ideation and intent. In this part, a threat to self-moderation (TSM) is a critical factor that turns defeat and humiliation into suicidal ideation [5]. Herein, the autobiographical memory bias is included as TSM, and may play a key role in the cognitive vulnerability to suicidal behavior [7–9]. Finally, the volitional phase governs the transition from suicidal ideation to suicide attempts. The planning, imagery, and past behavior of suicide are volitional moderators in this phase. Also, suicidal behavior can affect suicidal ideation in reverse [10]. Intriguingly, the emergence of suicidal ideation is a crucial part of the whole process of suicide, which could be effectively intervened. If someone is feeling trapped or distressed, interventions that target the factors within the motivational phase could reduce the likelihood of the emergence of suicidal ideation [11].

Over-general autobiographical memory (OGM) has been proven to be a major risk factor of suicidal ideation in several suicidal theories. In the IMV, a moderator in the motivational phase contributes to the production of suicidal ideation. In the cognitive model of suicidal behavior, the vulnerability factor activates the cognitive process of mental abnormality, suicidal ideation, and suicidal behavior [12]. A number of studies have shown that patients with a history of suicidal attempts had more OGM as compared to those without such history [9]. Thus, it could be inferred that OGM mediates the suicidal process by preventing the individuals from solving problems and envisioning the future by searching in the past experience, thereby creating a feeling of hopelessness and helplessness [13]. However, autobiographical memory is not a fixed entity with unchangeable specificity. As a type of cognitive training, memory specificity training (MeST) effectively reduces the generalization level of the autobiographical memory [14]. Therefore, as a moderator of suicidal ideation that can be intervened, OGM necessitates an in-depth investigation.

Notably, autobiographical memory is associated with past experiences. Experiencing trauma and abuse is a risk factor of OGM, which might serve as an adaptive function to limit the negative effects and distress associated with terrible recollections of the past [15]. The earlier the age of trauma, longer the duration and higher the severity of trauma. Childhood is the period when individuals gradually form coping styles and autobiographical memory extraction patterns. A previous study has shown that adults with a history of childhood trauma may have a problem in memory extraction; the earlier the trauma occurs, the higher the level of autobiographical memory generation [16].

Childhood trauma and OGM are two crucial risk factors of different suicidal phases. However, there is no clear evidence to show how they work together in the suicidal process and whether depression has any moderating effect. Herein, we hypothesized that OGM has different effects on the suicide process in depression patients and healthy individuals. The present study aimed to compare childhood trauma, OGM, suicidal ideation, and suicidal behavior between depression patients and healthy individuals and explore the differences caused by depression in suicidal pathways. Especially, the role of OGM in suicide process needs to be deduced for optimal interventions for different populations.

## Methods

### Participants

The current study comprised of 356 Chinese participants, including 180 depression patients and 176 healthy control individuals. The recruitment of depression patients in the depression group was carried out on-site via affiliated Hospitals of the Army Medical University and Chongqing Mental Health Center. The inclusion criteria were as follows: (a) diagnosed by both the Beck Depression Inventory second edition (BDI-II > 13) and clinical diagnosis (updated to meet the Diagnostic and Statistical Manual of Mental Disorders (DSM-5) [2013] criteria) of the major depressive disorder (MDD) by psychiatrists at hospitals; (b) no severe physical or other mental illnesses; (c) age between 18 and 65 years; (d) understanding the questionnaires. The majority of depression patients presented depression for > 1 year (*n* = 121; 67.2%), and reported administration of psychotropics (*n* = 99; 55%). The recruitment of healthy individuals in the control group was via online platform, according to the following inclusion criteria: (a) normal scores in the BDI-II (< 13) and clinical diagnosis (DSM-5); (b) no severe physical illness or mental illness; (c) age between 18 and 65 years; (d) understanding the questionnaires. The current study was approved by the Ethics Committee of the Army Medical University and Medical Ethics Committee of Chongqing Mental Health Center, China.

### Measures

#### Depressive symptoms

BDI-II [17] is a 21-item self-report survey assessing the severity of depressive symptoms over the past 2 weeks, using a four-point Likert scale of 0–3. The total score range was 0–63, according to the score boundaries provided by the original scale. It was categorized as 0–13 without depression, 14–19 with mild depression, 20–28 with moderate depression, and 29–63 with severe depression. The Chinese version of this measure had an alpha coefficient of 0.94 [18]. The current study found acceptable Cronbach’s α value (α = 0.95).

#### OGM

The OGM questionnaire (OGMQ) [19] is a 19-item self-report tool for the assessment of the specificity of autobiographical memory, using a four-point Likert scale (1 = perfect match; 4 = not match). An example of an item is as follows: “I can recall a specific thing that I experienced personally when I was in high school.” The total score can range was 19–76, and higher scores indicated a myriad of OGMs. This questionnaire was based on the autobiographical memory test (AMT) [8] and the sentence completion for events from the past test (SCEPT) [20]. It exhibited reliable internal consistency of the total scale (α = 0.87) in a healthy population of college students. The current study found acceptable value of Cronbach’s α (α = 0.94).

#### Childhood trauma

Childhood Trauma Questionnaire [21] is a 28-item self-report (CTQ-SF) measure assessing the maltreatment and trauma experience before 16 years of age, using a five-point Likert scale (1 = never; 5 = always). The five subscales included sexual abuse, physical abuse, emotional abuse, emotional neglect, and physical neglect. Each subscale contained 5 items, and the other 3 items were for testing the validity. The Chinese version of this measure is valid and reliable in healthy population [22]. The current study found a satisfactory internal consistency (α = 0.93).

#### Suicidal ideation

Beck scale for suicidal ideation-Chinese version (BSI-CV) [23] is a 19-item self-report questionnaire that evaluates the thoughts about life and death and the severity of suicidal ideation, using a three-point scale of 0–2. The total score can range from 0 to 38. If the score of item 4 or 5 is not 0, it indicates suicidal ideation. Higher scores indicate stronger suicidal ideation. Each question is asked about two time points: the current week and the worst point in the past. The BSI-CV was revised according to BSI [24], scale for suicidal ideation-current (SSI-C) [25], and scale for suicidal ideation-worst (SSI-W) [26]. An α-coefficient of 0.68–0.87 was estimated in adult community residents [23] and 0.95 in depression patients [27]. The data from Cronbach’s α were as follows: BSI-W(BSI-CV for worst-point suicidal ideation (WSI)) α = 0.97 and BSI-C (BSI-CV for current suicidal ideation (CSI)) α = 0.94 in the current study.

#### Previous suicidal behavior (PSB)

To measure the PSB, a 4-point scale was used (0 = never; 1 = once; 2 = twice; 3 = more than twice). The question, “how many times did you induce self-injury or suicidal behaviors, such as taking medicine or cutting your wrists in the past?” was addressed.

### Path model of suicidal ideation pathways

The path model of suicidal pathways was conceptualized based on previous studies and IMV, and the temporal ordering of the included variables. The path model was as follows: Childhood trauma is included in the pre-motivational phrase and presumed to influence the OGM, WSI and CSI in the motivational phrase directly. The OGM is presumed to influence both suicidal ideations. The WSI is considered to influence the CSI directly. It also affects PSB in the volitional phrase, which influences CSI in reverse.

### Data analysis

Descriptive and correlational analyses were conducted using IBM SPSS Statistics v.23 (IBM Co., Armonk, NY, USA). The descriptive statistics were performed to describe the demographic characteristics, depressive symptoms, suicidal ideation, and PSB of the participants. Cross-contingency tables (χ^2^) were used to compare the differences between the two groups. To explore the distribution of the data, preliminary data analyses, such as skewness, kurtosis, and multicollinearity were performed. An independent t-test was used to compare the scores from the questionnaires. Pearson’s product-moment correlation coefficients for variables were calculated to explore the associations between childhood trauma, OGM, WSI, CSI, and PSB.

Multigroup path analysis within a structural equation modeling framework was used to examine the moderating effect of depression and explore the differences in path models between depression and healthy groups. The analysis was carried out in four steps: First, the overall path model was examined. Second, an unconstrained model in which all parameters were allowed to differ across groups was established to confirm the same baseline model in groups. Third, differences between the baseline and constrained models were assessed by the χ^2^ test. The models were considered similar if the χ^2^ difference was nonsignificant [28]. Last, the direct and indirect path coefficients in the path model were estimated and compared between the groups.

To assess the overall model fit, the following goodness-of-fit measures and recommended cutoff points were applied [29]: Normed fit index (NFI), comparative fit index (CFI), and Tucker-Lewis index (TLI). If such indices were > 0.9, the model fit was interpreted as good, while < 0.85 indicated not acceptable. The absolute fit index including root mean squared error of approximation (RMSEA) was also used to assess the suitability of model verification. RMSEA indicated optimal suitability of the model if such an index was < 0.05 and acceptable if < 0.08. If χ^2^/df (normed chi-square) is < 5, the model is acceptable fit, and < 2 indicates good fit.

Furthermore, the maximum likelihood estimation method was chosen as it allows the estimation of all model path coefficients and can be utilized for computing fit statistics. The significance of the mediation effects was analyzed using a bootstrap procedure (2000 resamples) with 95% bias-corrected confidence interval (CI). An effect was considered significant at *P* < 0.05 if 0 is not included in the interval between the lower and upper thresholds [29]. The path models were estimated using the AMOS (Analysis of Moment Structure) software. All variables were treated as continuous, and both standardized and unstandardized estimates were obtained.

## Results

### Participant characteristics

The age of the participants was 29.5 ± 8.0-years-old in the depression group and 28.4 ± 8.8-years-old in the healthy group. No significant differences were detected in the age, gender, occupational status, annual household income, religion, nationality, and living style (*P* > 0.05) between the two groups. Participants in the depression group reported significantly higher levels of depressive symptoms as compared to healthy individuals (t = 24.42, *P* < 0.001). In the depression group, the ratios of CSI, WSI, and PSB were significantly much higher than those in healthy group (*P* < 0.001). The results are reported in Table 1.

**Table 1:** Demographics of depression and healthy groups

Sample characteristics	Depression (*N* = 180)	Healthy (*N* = 176)	χ^2^	*P*
Mean age ± SD (years)	29.5 ± 8	28.4 ± 8.8	1.236 (t)	0.217
Gender			2.85	0.091
Male	78 (43.3)	92 (52.3)		
Female	102 (56.7)	84 (47.7)		
Occupational status			3.26	0.071
Yes	104 (57.8)	118 (67)		
No	76 (42.2)	58 (33)		
Annual household income (10,000 Yuan)			0.25	0.881
< 5	37 (20.6)	33 (18.8)		
5–20	135 (75)	136 (77.3)		
> 20	8 (4.4)	7 (4)		
No religion	170 (94.4)	168 (95.5)	0.19	0.664
Han nationality	169 (93.9)	166 (94.3)	0.03	0.864
Live alone	23 (12.8)	14 (8)	2.22	0.136
BDI-II (Mean ± SD)	24.01 ± 10.31	3.89 ± 3.95	24.42 (t)^***^	< 0.001
CSI			43.71^***^	< 0.001
Yes	55 (30.6)	7 (4)		
No	125 (69.4)	169 (96)		
WSI			91.38^***^	< 0.001
Yes	122 (67.8)	31 (17.6)		
No	58 (32.2)	145 (82.4)		
PSB			42.36^***^	< 0.001
Yes	50 (27.8)	5 (2.8)		
No	130 (72.2)	171 (97.2)		

Note. Values are expressed as a number (%); t: Independent t-test t-value. BDI-II: Beck Depression Inventory second edition; ^***^P < 0.001.*WSI* Worst-point suicidal ideation, *CSI* Current suicidal ideation, *PSB* Previous suicidal behavior

### Preliminary data analyses and comparisons

As shown in Table 2, the distribution of the variables was similar to the normality as the absolute value of skewness was < 3, and the absolute value of kurtosis was < 8–10. Also, the multicollinearities between OGM, childhood trauma, PSB, WSI, and CSI were not critical (variance inflation factor,VIF = 1.30, 1.31, 1.75, 2.16 < 5) [29]. The individuals in the depression group had significantly higher mean scores on all the measures as compared to the healthy individuals.

**Table 2:** Descriptive statistics, t-test, normal distribution of variables

Measures	Mean	SD	Skewness	Kurtosis	Depression Mean (SD)	Healthy Mean (SD)	*t*	*P*
CT	42.1	14.86	0.77	−0.29	49.59 (14.69)	34.44 (10.51)	11.20^***^	< 0.001
OGM	36.39	12.39	0.45	−0.31	42.55 (11.55)	30.09 (9.80)	10.96^***^	< 0.001
WSI	7.56	10.09	1.1	−0.16	12.94 (10.85)	2.06 (5.11)	12.15^***^	< 0.001
CSI	2.17	5.24	2.81	7.59	3.82 (6.51)	0.49 (2.58)	6.38^***^	< 0.001
PSB	0.29	0.75	2.59	5.65	0.52 (0.94)	0.05 (0.34)	6.33^***^	< 0.001

Note: *CT* Childhood trauma, *OGM* Over-general autobiographical memory, *WSI* Worst-point suicidal ideation, *CSI* Current suicidal ideation, *PSB* Previous suicidal behavior, *SD* Standard deviation. ^***^P < 0.001

### Correlation between variables

The results of group-specific correlations between all the variables were included in the path model (Table 3). In depression group, all the correlations between variables were significant except those between OGM and PSB. In the healthy group, childhood trauma was significantly correlated with suicidal ideations, OGM was significantly correlated with WSI and PSB, and WSI was significantly correlated with PSB and CSI.

**Table 3:** Correlations between groups of variables

Variables	Depression				Healthy			
	CT	OGM	WSI	PSB	CT	OGM	WSI	PSB
CT	–				–			
OGM	0.208^**^	–			0.049	–		
WSI	0.202^**^	0.214^**^	–		0.342^**^	0.235^**^	–	
PSB	0.225^**^	0.035	0.612^**^	–	0.063	0.184^*^	0.544^**^	–
CSI	0.214^**^	0.384^**^	0.479^**^	0.340^**^	0.385^**^	0.098	0.367^**^	−0.016

Note: *CT* Childhood trauma, *OGM* Over-general autobiographical memory, *WSI* Worst-point suicidal ideation, *CSI* Current suicidal ideation, *PSB* Previous suicidal behavior. ^*^P < 0.05, ^**^P < 0.01

### Full path model analysis

**Fig. 1:**
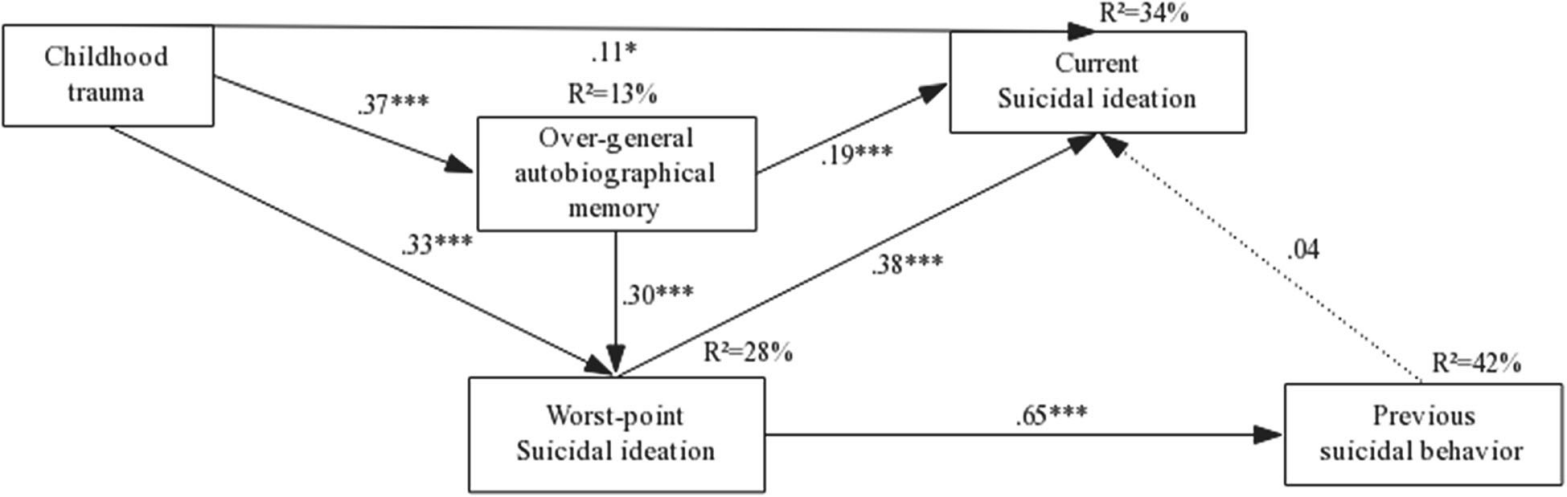
Path model (*n* = 356). Standardized path coefficients among variables are presented. Dotted line represents nonsignificant path coefficients. ^*^P < 0.05; ^***^P < 0.001

The current results showed that the suicidal path model presented an overall acceptable fit with the observed data (χ^2^/df = 2.127; CFI = 1.00; TLI = 0.98; NFI = 0.99; RMSEA = 0.056; *P* = 0.12). Figure 1 illustrates the significance tests of the hypothesis for the proposed path model. R-square (R^2^) is the coefficient of determination that indicates the proportion of the variance for endogenous variable in order to assess the power of path model. OGM and WSI mediated the correlation between childhood trauma and CSI (*P* < 0.05). The indirect effect was 70.28% of the total effect.

### Multigroup comparison and the moderating effect of depression

Since the baseline models for each group were the same, multigroup analysis was conducted. First, a multigroup analysis with the unconstrained model showed an acceptable baseline model for both healthy and depression groups (χ^2^/df = 3.025; *P* = 0.016; CFI = 0.97; TLI = 0.87; NFI =0.96; RMSEA = 0.076). Then, the constrained model with equal intercepts was established between the groups and examined to assess the moderating effect of depression. The results revealed that the constrained model was not acceptable (χ^2^/df = 4.418; *P* < 0.001; CFI = 0.87; TLI = 0.78; NFI =0.84; RMSEA = 0.098). Also, the χ^2^ difference test showed a significant difference between the base model and constrained model (χ^2^(8) = 40.81, *P* < 0.05), suggesting that depression plays a critical role in the moderating effect. The path models showed differences between the depression and healthy groups.

In this study, depression moderates the direct effects between WSI and PSB, OGM and CSI, as well as, PSB and CSI as illustrated in Table 4 with significant z scores (*P* < 0.05). Depression patients with WSI were more likely to exhibit suicidal behavior. OGM had a significant positive effect on CSI only in the depression group, while PSB had a significantly negative effect on CSI only in the healthy group. The indirect path, childhood trauma→OGM → CSI, also showed a significant difference between the two groups (z-score: -2.342^*^, *P* < 0.05). Childhood trauma had a positive effect, 43.26% of the total indirect effect, on CSI through OGM in the depression group (β = 0.062, 95% CI: 0.023–0.117; *P* = 0.002). However, this path was not significant in the healthy group (β = 0.002; 95% CI: − 0.005–0.017; *P* = 0.337).

**Table 4:** Parameter estimation between groups of path model

Path	Depression				Healthy				z score
	β	B	SE	*P*	β	B	SE	*P*	
CT → OGM	0.208^**^	0.164	0.057	0.004	0.049	0.046	0.07	0.516	−1.298
OGM → WSI	0.18^*^	0.169	0.069	0.014	0.218^**^	0.114	0.036	0.002	−0.711
CT → WSI	0.165^*^	0.122	0.054	0.025	0.332^***^	0.161	0.034	< 0.001	0.616
WSI → PSB	0.612^***^	0.053	0.005	< 0.001	0.544^***^	0.036	0.004	< 0.001	−2.467^*^
OGM → CSI	0.295^***^	0.167	0.036	< 0.001	0.037	0.01	0.018	0.58	−3.924^**^
WSI → CSI	0.335^***^	0.201	0.048	< 0.001	0.416^***^	0.208	0.042	< 0.001	0.114
PSB → CSI	0.111	0.77	0.54	0.154	−0.265^***^	−1.98	0.583	< 0.001	−3.461^**^
CT → CSI	0.059	0.026	0.028	0.352	0.261^***^	0.063	0.017	< 0.001	1.13

Note. *CT* Childhood trauma, *OGM* Over-general autobiographical memory, *WSI* Worst-point suicidal ideation, *CSI* Current suicidal ideation, *PSB* Previous suicidal behavior, *β* Standardized beta, *B* Unstandardized estimate, *SE* Standard error. ^*^P < 0.05, ^**^P < 0.01, ^***^P < 0.001

## Discussion

To the best of our knowledge, this is the first study that connects CSI and WSI of depressed and healthy control groups with suicidal behavior, childhood trauma, and autobiographical memory together. The variables belong to different phases of suicide and constitute a complete timeline of suicide. These results suggested that the suicidal ideation and behavior of depression patients are significantly higher than those of healthy individuals, while the background factors (childhood trauma) and the moderator (OGM) of suicide are more severe in depression patients. The multigroup path analysis demonstrated that only in depression patients, OGM significantly affects the CSI and acts as an intermediary between childhood trauma and CSI. However, there was no significant effect of OGM on CSI in healthy individuals, indicating that OGM plays different roles in the emergence of suicidal ideation in different populations.

Childhood trauma as a risk factor of depression [30] and OGM as a stable trait of depression [31] were obviously more severe in the depression group than the healthy control. Also, the incidence of suicidal ideation was much higher in depressed people, about 4–8 times, as compared to that in healthy people. The rate of suicide attempts in depression patients is 10-fold higher than that in healthy people. These statistics were similar to the data enumerated by WHO [32]. Patients with depression are designated as a high-risk population for suicide due to the substantial health disparities [33]. Previous studies have shown that the structural and functional abnormalities of the brain in depression might be the physiological basis of suicide [34–36]. Based on the results of this study, childhood trauma and OGM may be the psychological factors leading to suicide.

The results from correlation analyses showed that childhood trauma and OGM were correlated with WSI, indicating that they are critical factors of suicidal ideation according to the IMV model [4]. PSB was strongly correlated to WSI, which was consistent with a previous study, wherein high intensity of WSI increased the risk of suicide attempts [37]. In the case of CSI, a strong correlation with WSI was observed than PSB in the depression group, and only significantly correlated with WSI in the healthy group. Nam et al. [38] confirmed that WSI might be an independent predictor of follow-up suicidal ideation intensity.

Surprisingly, childhood trauma had a positive correlation with OGM in depression patients, but no significant correlation of these factors was observed in healthy people. The results of CTQ suggested that depression patients suffered more severe childhood trauma than healthy people. Childhood trauma is a significant risk factor for developing MDD in later life [39]. In addition, higher intensity of childhood trauma was associated with stronger OGM [40, 41]. It was verified that childhood abuse predicted greater rumination and impaired executive function and maladaptive emotion regulation, which were causes of OGM [42–44]. However, in healthy individuals, OGM is sensitive to the age effect [45]. Low severity or less experience of childhood trauma might not have an impact on OGM in healthy people, which might explain this phenomenon.

The data from path analysis confirmed our theoretical assumptions that OGM and WSI mediated the correlation between childhood trauma and CSI. The indirect effect was stronger than the direct effect. However, depression had a moderating effect on the path model. Only in the depression group, OGM had a positive correlation with CSI and served as a mediator between childhood trauma and CSI. OGM is a stable trait of depression and belongs to the negative cognitive processing bias, which is hard to recover from depression [15]. The negative cognitive processing bias of depression patients plays a critical role in maintaining negative emotional state and obtaining suicide-related information that activates suicide schemata and increases the possibility of suicide [46]. As a cognitive defect, OGM constantly contributes to the suicidal ideation in depression patients. However, the autobiographical memory of healthy people does not become a severely damaged cognitive factor like that in depression patients. A previous study suggested that healthy people with suicidal attempt were likely to recall overgeneralized memories as compared to those with only suicidal ideation [47]. In the current study, WSI was significantly correlated with the suicidal attempt in healthy people, which might be an explanation for these results. Another finding was that PSB was negatively correlated with CSI and less affected by WSI in the healthy group. One reason might be that most proportion of suicides in healthy people are impulsive attempts [48], which do not follow the depression-hopelessness path to suicidal behavior [49], with lower expectations of death and suicidal ideation [50].

These results suggested that improving the specificity of autobiographical memory may be an effective way to prevent suicidal ideation for depression. Life-review therapy [51] and MeST [52] aimed to recall memories in detail. These were effective and applied as a widely-used cognitive training for OGM [53]; it could efficiently prevent WSI. These might be more useful in preventing CSI in depression patients than healthy individuals. In the case of healthy individuals, we should find other factors that could moderate CSI directly.

Nevertheless, the present study has several limitations. First, the number of samples was not sufficient, especially the number of individuals with a history of suicide attempts in the healthy population was extremely small, which might provide inaccurate results of data analysis. Thus, these conclusions need to be confirmed in a larger sample. Moreover, since this was a cross-sectional study, the retrospective data collection may deviate from the real situation. Thus, prospective studies are essential to verify the conclusions in the future. Furthermore, suicide is a complex process affected by biological factors, mental disorders, and psychological factors; OGM considered in this study is only one of the psychological factors. Hence, the conclusions of this study can only be used as one of the explanations, and the corresponding brain mechanism needs to be explored further.

## Conclusions

This study identified the different roles of OGM in the suicidal ideation of depressed and healthy people. In depression patients, it affects the CSI and WSI and mediates the CSI due to the effect of childhood trauma. In healthy people, it can only affect the WSI. As an adjustable risk factor, the autobiographical memory might be a target of intervention for suicidal ideation in depression patients. Since training in specific memory retrieval has been proven to be effective in depression, future studies should consider whether it can reduce the emergence of suicidal ideation and suicidal behavior.

## Supplementary information

**Additional file 1:.** Psychological questionnaire.

## Data Availability

The datasets used and/or analyzed during the current study are available from the corresponding author upon reasonable request.

## Abbreviations

OGM: Over-general autobiographical memory
CSI: Current suicidal ideation
WSI: Worst-point suicidal ideation
IMV: Integrated motivational-volitional
TSM: Threat to self-moderation
DSM-5: Diagnostic and statistical manual of mental disorders
MDD: Major depressive disorder
OGMQ: OGM questionnaire
SCEPT: Sentence completion for events from the past test
BSI-CV: Beck scale for suicidal ideation-Chinese version
SSI-C: Scale for suicidal ideation-current
SSI-W: Scale for suicidal ideation-worst
PSB: Previous suicidal behavior
AMOS: Analysis of moment structure
VIF: Variance inflation factor
