# Supplementary material for: Distinct effects of over-general autobiographical memory on suicidal ideation among depressed and healthy people
Source: BMC Psychiatry. 2020 Oct 12;20:501. doi: 10.1186/s12888-020-02877-6 (PMC7549224; doi:10.1186/s12888-020-02877-6)
Supplement: Supplementary file 1 — Additional file 1:. Psychological questionnaire. [file 12888_2020_2877_MOESM1_ESM.docx]

**Psychological questionnaire**

Dear friend:

Hello! You are welcome to participate in one of our psychological surveys. In order to ensure the scientific nature of the investigation, please pay attention to the following aspects when answering the questions:

1. This test is only for scientific research and has no other purpose, so there is no need to worry about it.

2. there is no time limit for this test, but there is no need to take more time to think about the test questions over and over again.

**3. be sure to answer each test question, do not miss the question, thank you for your cooperation!**

**Basic information**

1. Name :______________________.

2. Gender :○ male ○ female

3. Nationality :○ Han nationality ○ other ethnic _________________

4. Age: year-old

5. Current occupation :○students ○ farmers, fishermen, herdsmen ○ administrators ○professional and technical personnel ○private owners ○ service personnel ○temporary job ○soldier ○unemployed, retired ○housewives ○other _________________

6. Residence: ○ living alone ○ dormitory ○ shared residence with family

○ other _________________

7. Family financial situation (annual household income): ○rich(>200,000yuan) ○normal(5-200,000yuan) ○poor (<50,000yuan)

8. Religious beliefs :○ none ○ Catholic ○ Christian ○ Buddhist ○ Taoism ○ Islam

○ other _________________

1. **BDI-2**

Instructions: This questionnaire consists of 21 groups of statements. Please read each group of statements carefully. And then pick out the one statement in each group that best describes the way you have been feeling during the past two weeks, including today. Circle the number beside the statement you have picked. If several statements in the group seem to apply equally well, circle the highest number for that group. Be sure that you do not choose more than one statement for any group, including Item 16 (Changes in Sleeping Pattern) or Item 18 (Changes in Appetite).

| 1. Sadness   I do not feel sad.  I feel sad much of the time.  I am sad all the time.  3. I am so sad or unhappy that I can't stand it. | 6. Punishment Feelings  0. I don't feel I am being punished.  1. I feel I may be punished.  2. I expect to be punished.  3. I feel I am being punished. |  |
| --- | --- | --- |
| 2.Pessimism  I am not discouraged about my future.  1. I feel more discouraged about my future than I used to.  2.I do not expect things to work out for me.  3. I feel my future is hopeless and will only get worse. | 7. Self-Dislike  0. I feel the same about myself as ever.  1. I have lost confidence in myself.  2. I am disappointed in myself.  3. I dislike myself. |  |
| 3.Past Failure  I do not feel like a failure.  I have failed more than I should have.  2. As I look back, I see a lot of failures.  3. I feel I am a total failure as a person. | 1. Self-Criticalness   I don't criticize or blame myself more than usual.  I am more critical of myself than I used to be.  2. I criticize myself for all of my faults.  3. I blame myself for everything bad that happens. |  |
| 4.Loss of Pleasure  0. I get as much pleasure as I ever did from the things I enjoy.  1. I don't enjoy things as much as I used to.  2. I get very little pleasure from the things I used to enjoy.  3. I can't get any pleasure from the things I used to enjoy. | 9. Suicidal Thoughts or Wishes  0. I don't have any thoughts of killing myself.  1. I have thoughts of killing myself, but I would not carry them out.  2. I would like to kill myself.  3. I would kill myself if I had the chance. |  |
| 5. Guilty Feelings  0. I don't feel particularly guilty.  1. I feel guilty over many things I have done or should have done.  2. I feel quite guilty most of the time.  3. I feel guilty all of the time | 10. Crying  0. I don't cry anymore than I used to.  1. I cry more than I used to.  2. I cry over every little thing.  3. I feel like crying, but I can't. |  |
| 1. Agitation   I am no more restless or wound up than usual.  I feel more restless or wound up than usual.  2. I am so restless or agitated, it's hard to stay still.  3. I am so restless or agitated that I have to keep moving or doing something. | 17. Irritability  0. I am not more irritable than usual.  1. I am more irritable than usual.  2. I am much more irritable than usual.  3. I am irritable all the time. | |
| 12. Loss of Interest  0. I have not lost interest in other people or activities.  1. I am less interested in other people or things than before.  2. I have lost most of my interest in other people or things.  3. It's hard to get interested in anything. | 18. Changes in Appetite  0. I have not experienced any change in my appetite.  1a My appetite is somewhat less than usual.  1b My appetite is somewhat greater than usual.  2a My appetite is much less than before. 2b My appetite is much greater than usual.  3a I have no appetite at all.  3b I crave food all the time. | |
| 13.Indecisiveness  I make decisions about as well as ever.  I find it more difficult to make decisions than usual.  2.I have much greater difficulty in making decisions than I used to.  3. I have trouble making any decisions. | 19. Concentration Difficulty  0. I can concentrate as well as ever.  1. I can't concentrate as well as usual.  2. It's hard to keep my mind on anything for very long.  3. I find I can't concentrate on anything. | |
| 1. Worthlessness   I do not feel I am worthless.  I don't consider myself as worthwhile and useful as I used to.  2.I feel more worthless as compared to others.  3. I feel utterly worthless. | 20. Tiredness or Fatigue  0. I am no more tired or fatigued than usual.  1. I get more tired or fatigued more easily than usual.  2. I am too tired or fatigued to do a lot of the things I used to do.  3. I am too tired or fatigued to do most of the things I used to do. | |
| 1. Loss of Energy   I have as much energy as ever.  I have less energy than I used to have.  2. I don't have enough energy to do very much. 3. I don't have enough energy to do anything. | 1. Loss of Interest in Sex   I have not noticed any recent change in my interest in sex.  I am less interested in sex than I used to be.  2.I am much less interested in sex now.  3. I have lost interest in sex completely. | |
| 1. Changes in Sleeping Pattern   I have not experienced any change in my sleeping.  1a I sleep somewhat more than usual.  1b I sleep somewhat less than usual.  2a I sleep a lot more than usual.  2b I sleep a lot less than usual.  3a I sleep most of the day.  3b I wake up 1-2 hours early and can't get back to sleep. |  | |

1. **OGMQ**

Guide: Please read carefully and fully understand each of the following items, answer the following questions according to your actual situation, and type "√" under the fits your situation (check only one option for each question)(where 1 represents full conformity ,2 indicates comparative conformity ,3 indicates comparative non-conformity, and 4 indicates complete non-conformity).

| **Project** |  |  |  |  |
| --- | --- | --- | --- | --- |
| 1. I am able to recall a specific experience that I had experienced in high school. | 1 | 2 | 3 | 4 |
| 1. I can clearly recall a specific feeling of disgust at something. | 1 | 2 | 3 | 4 |
| 1. I can clearly remember the expression on the other person's face when giving help. | 1 | 2 | 3 | 4 |
| 1. I can recall a particular time I went to a bookstore. | 1 | 2 | 3 | 4 |
| 1. I can clearly recall the feeling of happiness and excitement when I was reunited with my family. | 1 | 2 | 3 | 4 |
| 1. When I recall a sad personal experience, I can overcome the irrelevant memories that appear in my brain to ensure a smooth memory. | 1 | 2 | 3 | 4 |
| 1. I am able to recall a specific thing that I experienced personally during the travel period (more than 1 day) | 1 | 2 | 3 | 4 |
| 1. I can clearly remember the exact location of a trip. | 1 | 2 | 3 | 4 |
| 1. I am able to recall a clear picture of a physical injury. | 1 | 2 | 3 | 4 |
| 1. I can clearly recall the feeling of surprise and happiness when I received a gift. | 1 | 2 | 3 | 4 |
| 1. When I recall a painful personal experience, I can correct the wrong memories in my brain to ensure smooth memories. | 1 | 2 | 3 | 4 |
| 1. I am able to recall a specific scenario in which I failed at work (or study, activity, etc.). | 1 | 2 | 3 | 4 |
| 1. I can recall the specific scenes that occurred during a recent (1 week to 3 months) party. | 1 | 2 | 3 | 4 |
| 1. I can remember exactly where I once ate a meal. | 1 | 2 | 3 | 4 |
| 1. I can clearly recall the feeling of regret when I missed the opportunity. | 1 | 2 | 3 | 4 |
| 1. When I recall personal experiences that have made me warm, I can always recall something warm. | 1 | 2 | 3 | 4 |
| 1. When I recall a fearful personal experience, I can identify the uncertain memories in my brain to ensure a smooth memory. | 1 | 2 | 3 | 4 |
| 1. I can recall a clear picture of a moment that makes me feel very quiet and comfortable. | 1 | 2 | 3 | 4 |
| 1. I can clearly recall the painful feeling of a physical injury. | 1 | 2 | 3 | 4 |

1. **BSI-CV**

The following items are some questions about your thoughts on life and death. Each question asks you how you felt in the last week and how you felt in the past when you were most depressed. If the phenomenon occurs only in the last week, when filling in the corresponding item "the most depressed time ", it should be written according to the situation of the last week. The answers to each question are different, please pay attention to see the questions and alternative answers, and then according to your situation to choose the most appropriate answer.

| **1. how much do you want to live?** | | | | | | |
| --- | --- | --- | --- | --- | --- | --- |
| **Last week** | Medium to strong | | Weak | | No desire to live | |
| **The most depressed time** | Medium to strong | | Weak | | No desire to live | |
| **2.How much do you want to die?** | | | | | | |
| **Last week** | No desire to die | | Weak | | Medium to strong | |
| **The most depressed time** | No desire to die | | Weak | | Medium to strong | |
| **3. Are your reasons for living better than your reasons for dying?** | | | | | | |
| **Last week** | To live is better than to die | | Both are equal | | To die is better than to live | |
| **The most depressed time** | To live is better than to die | | Both are equal | | To die is better than to live | |
| **4.How much do your desire to commit suicide?** | | | | | | |
| **Last week** | No | | Weak | | Medium to strong | |
| **The most depressed time** | No | | Weak | | Medium to strong | |
| **5. How much do you want external forces to end your life, that is, have a "passive suicide desire"? (For example, I hope to stay asleep and not wake up, die unexpectedly, etc.)** | | | | | | |
| **Last week** | No | | Weak | | Medium to strong | |
| **The most depressed time** | No | | Weak | | Medium to strong | |
| **If the answer to item 4 or 5 above is "weak" or "medium to strong ", whether for the" Last week "or" The most depressed time", continue to ask the next question; otherwise, please jump to item 20.** | | | | | | |
| **6. how long does your suicidal thought last?** | | | | | | |
| **Last week** | Short, fleeting | Long duration | | continuous or almost continuous | | No suicidal thoughts in the last week |
| **The most depressed time** | Short, fleeting | Long duration | | continuous or almost continuous | |  |
| **7. how often do you think of suicide?** | | | | | | |
| **Last week** | Rarely, occasionally | Sometimes | | Often or continuously | | No suicidal thoughts in the last week |
| **The most depressed time** | Rarely, occasionally | Sometimes | | Often or continuously | |  |
| **8. What is your attitude towards suicide?** | | | | | | |
| **Last week** | Rejection | | Contradiction or indifference | | Acceptance | |
| **The most depressed time** | Rejection | | Contradiction or indifference | | Acceptance | |
| **9. What do you think of your ability to control suicidal thoughts and not turn them into actions?** | | | | | | |
| **Last week** | Can control | | I don't know if I can control | | Can’t control | |
| **The most depressed time** | Can control | | I don't know if I can control | | Can’t control | |
| \| **10. If suicidal thoughts appear, to what extent can certain concerns (such as taking care of family members, irreversible death, etc.) prevent you from committing suicide?** \| \| \| \| \| \| \| \| \| \| \| \| \| \| --- \| --- \| --- \| --- \| --- \| --- \| --- \| --- \| --- \| --- \| --- \| --- \| --- \| \| **Last week** \| Stop suicide \| \| \| \| Reduce the risk of suicide \| \| \| \| \| No concern or impact \| \| \| \| **The most depressed time** \| Stop suicide \| \| \| \| Reduce the risk of suicide \| \| \| \| \| No concern or impact \| \| \| \| 1. **When you want to commit suicide, what is the main reason?** \| \| \| \| \| \| \| \| \| \| \| \| \| \| **Last week** \| Control the situation,  Seek attention and revenge \| Escape, alleviate suffering,  Solve problems \| \| \| \| \| In both cases \| \| \| \| No suicidal thoughts in the last week \| \| \| **The most depressed time** \| Control the situation,  Seek attention and revenge \| Escape, alleviate suffering,  Solve problems \| \| \| \| \| In both cases \| \| \| \|  \| \| \| **12. Have you thought about ways to end your life?** \| \| \| \| \| \| \| \| \| \| \| \| \| \| **Last week** \| Never thought \| \| Yes, but no details \| \| \| \| \| Draw up specific details or plans \| \| \| \| \| \| **The most depressed time** \| Never thought \| \| Yes, but no details \| \| \| \| \| Draw up specific details or plans \| \| \| \| \| \| **13. What are the conditions or opportunities for you to implement your suicidal thoughts?** \| \| \| \| \| \| \| \| \| \| \| \| \| \| **Last week** \| No ready method,  no opportunity \| \| Need time or effort to prepare suicide instruments \| \| \| Available methods and opportunities or expected future ones \| \| \| \| \| \| No suicidal thoughts in the last week \| \| **The most depressed time** \| No ready method,  no opportunity \| \| Need time or effort to prepare suicide instruments \| \| \| Available methods and opportunities or expected future ones \| \| \| \| \| \|  \| \| 1. **Do you believe in your ability and courage to commit suicide?** \| \| \| \| \| \| \| \| \| \| \| \| \| \| **Last week** \| Without courage, too weak,  Fear, inability \| \| \| Not sure if I’m capable, courageous \| \| \| \| \| Be confident of my ability and courage \| \| \| \| \| **The most depressed time** \| Without courage, too weak,  Fear, inability \| \| \| Not sure if I’m capable, courageous \| \| \| \| \| Be confident of my ability and courage \| \| \| \| \| 1. **Do you really expect to try suicide at some point?** \| \| \| \| \| \| \| \| \| \| \| \| \| \| **Last week** \| No \| \| \| Uncertain \| \| \| \| \| Yes \| \| \| \| \| **The most depressed time** \| No \| \| \| Uncertain \| \| \| \| \| Yes \| \| \| \| \| **16. How was your preparation for suicide?** \| \| \| \| \| \| \| \| \| \| \| \| \| \| **Last week** \| Not ready \| \| \| Partially completed  (e.g. start collecting tablets) \| \| \| \| \| complete (e.g., pills,  blade, bullet gun) \| \| \| \| \| **The most depressed time** \| Not ready \| \| \| Partially completed  (e.g. start collecting tablets) \| \| \| \| \| complete (e.g., pills,  blade, bullet gun) \| \| \| \| \| 1. **Have you started writing your suicide note?** \| \| \| \| \| \| \| \| \| \| \| \| \| \| **Last week** \| No consideration \| \| \| Just think, start, not finish \| \| \| \| \| Finish writing \| \| \| \| \| **The most depressed time** \| No consideration \| \| \| Just think, start, not finish \| \| \| \| \| Finish writing \| \| \| \| \| **18. Do you have something to deal with because you expect to end your life? such as buying insurance or preparing a will.** \| \| \| \| \| \| \| \| \| \| \| \| \| \| **Last week** \| No \| \| \| Considered or made some arrangements \| \| \| \| \| Have a certain plan or arrangement completed \| \| \| \| \| **The most depressed time** \| No \| \| \| Considered or made some arrangements \| \| \| \| \| Have a certain plan or arrangement completed \| \| \| \| \| **19. Do you let people know your suicidal thoughts?** \| \| \| \| \| \| \| \| \| \| \| \| \| \| **Last week** \| Be frank and open \| Don't take the initiative \| \| \| \| \| Try to cheat or conceal \| \| \| \| No suicidal thoughts in the last week \| \| \| **The most depressed time** \| Be frank and open \| Don't take the initiative \| \| \| \| \| Try to cheat or conceal \| \| \| \|  \| \| | | | | | | |

1. **How many times did you induce self-injury or suicidal behaviors, such as taking medicine or cutting your wrists in the past?**

○ 0 = never; ○1 = once; ○2 = twice; ○3 = more than twice

4. **CTQ-SF**

Guide: This questionnaire is about your childhood (before age 16) growth experience. According to your experience at the time, please choose the answer that best suits your situation from the five options of "1 = never true, 2 = rarely true, 3 = sometimes true, 4 = often true, 5 = very often true", if you are not very clear, please try to estimate.

**Before the age of 16:**

| 1. I didn’t have enough to eat. | 1 | 2 | 3 | 4 | 5 |
| --- | --- | --- | --- | --- | --- |
| 2. I knew that there was someone to take care of me and protect me. | 1 | 2 | 3 | 4 | 5 |
| 3.People in my family called me things like “stupid,””lazy,”or “ugly.” | 1 | 2 | 3 | 4 | 5 |
| 4. My parents were too drunk or high to take care of the family. | 1 | 2 | 3 | 4 | 5 |
| 5. There was someone in my family who helped me feel that I was important or special. | 1 | 2 | 3 | 4 | 5 |
| 6. I had to wear dirty clothes. | 1 | 2 | 3 | 4 | 5 |
| 7. I felt loved. | 1 | 2 | 3 | 4 | 5 |
| 8. I thought that my parents wished I had never been born. | 1 | 2 | 3 | 4 | 5 |
| 9. I got hit so hard by someone in my family that I had to see a doctor or go to the hospital. | 1 | 2 | 3 | 4 | 5 |
| 10. The situation in my home at the time needed improvement. | 1 | 2 | 3 | 4 | 5 |
| 11. People in my family hit me so hard that it left me with bruises or marks. | 1 | 2 | 3 | 4 | 5 |
| 12. I was punished with a belt, a board, a cord, or some other hard object. | 1 | 2 | 3 | 4 | 5 |
| 13. People in my family looked out for each other. | 1 | 2 | 3 | 4 | 5 |
| 1. People in my family said hurtful or insulting things to me. | 1 | 2 | 3 | 4 | 5 |
| 15. I believe that I was physically abused. | 1 | 2 | 3 | 4 | 5 |
| 16.I had the perfect childhood. | 1 | 2 | 3 | 4 | 5 |
| 17. I got hit or beaten so badly that it was noticed by someone like a teacher, neighbour, or doctor. | 1 | 2 | 3 | 4 | 5 |
| 18. I felt that someone in my family hated me. | 1 | 2 | 3 | 4 | 5 |
| 19. People in my family felt close to each other. | 1 | 2 | 3 | 4 | 5 |
| 20. Someone tried to touch me in a sexual way, or tried to make me touch them. | 1 | 2 | 3 | 4 | 5 |
| 21. Someone threatened to hurt me or tell lies about me unless I did something sexual with them. | 1 | 2 | 3 | 4 | 5 |
| 22. I had the best family in the world. | 1 | 2 | 3 | 4 | 5 |
| 23. Someone tried to make me do sexual things or watch sexual things. | 1 | 2 | 3 | 4 | 5 |
| 24. Someone molested me. | 1 | 2 | 3 | 4 | 5 |
| 1. I believe I was emotionally abused. | 1 | 2 | 3 | 4 | 5 |
| 26. There was someone to take me to the doctor if I needed it. | 1 | 2 | 3 | 4 | 5 |
| 27. I believe that I was sexually abused. | 1 | 2 | 3 | 4 | 5 |
| 28. My family was a source of strength and support. | 1 | 2 | 3 | 4 | 5 |
| How old are you when you first have sex in your life _______ years old (if never ,"88") | | | | | |
